# Advances in Biosensor Technology for Illicit Drug Detection Enable Effective Wastewater Surveillance

**DOI:** 10.1021/cbe.4c00188

**Published:** 2025-10-21

**Authors:** Qihang Xu, Ying Guo, Gang Niu, Heping Wu, Frederic Coulon, Zhugen Yang

**Affiliations:** † Faculty of Engineering and Applied Sciences, 2717 Cranfield University, Cranfield MK43 0AL, U.K.; ‡ School of Electronic Science and Engineering, Key Laboratory of the Ministry of Education & International Center for Dielectric Research, Electronic Materials Research Laboratory, 12480 Xian Jiaotong University, Xian 710049, China

**Keywords:** illicit drug, biosensor, public health, wastewater-based epidemiology, real-time monitoring, point-of-need

## Abstract

Drug abuse has been a global public health challenge, requiring robust and timely strategies for monitoring drug consumption at the community level. Wastewater-based epidemiology (WBE) offers population-scale insights by analyzing drug biomarkers in sewage, yet conventional analytical methods can be time-consuming and resource-intensive. Biosensor technology is rapidly emerging as a promising tool for detecting illicit drugs in complex wastewater matrices, offering significant advantages in speed, specificity, and cost-effectiveness over conventional analytical methods. This review explores the emerging role of biosensor technology in enhancing WBE for illicit drug detection. We first outline the principles of WBE and discuss conventional analytical techniques used in wastewater drug surveillance, noting their limitations in cost, throughput, and real-time applicability. Next, we examine key biosensor platforms encompassing electrochemical, optical, and other transducer-based designs and highlight their capacity to rapidly and selectively detect target drugs or metabolites in complex wastewater matrics. We then address the principal challenges of biosensor deployment in WBE, including sample matrix interference, sensor fouling, and the need for calibration and standardization. Finally, we identify critical research gaps, such as further miniaturization, multiplexed detection, and integration with Internet of Things (IoT) and big data analytics. By merging biosensor innovation with WBE, this multidisciplinary approach promises more efficient, adaptable, and community-focused solutions for tracking illicit drug trends and informing public health policy.

## Graphical abstract

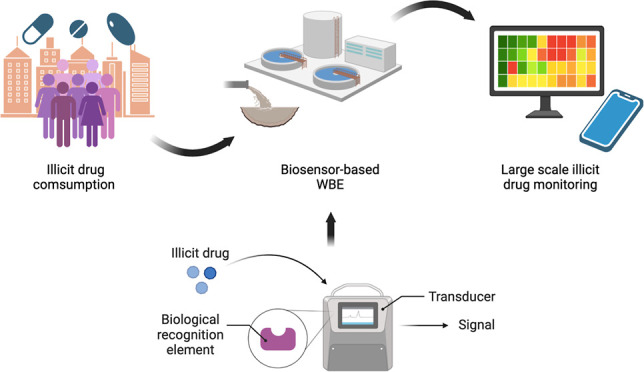

## 1 Introduction

Illicit drugs and their metabolites are widely detected in the environment, posing significant public health and safety concerns including rising addiction rates, overdose incidents, spread of communicable diseases, and increased economic burdens. Driven by the stringent needs of drug regulators and policymakers, there is an urgent requirement for a reliable and effective detection methodology that can accurately identify and monitor illicit drugs and/or their metabolites, which should facilitate timely data to inform public health interventions and guide evidence-based policymaking. According to the United Nations World Drug Report 2021, common drugs like amphetamines (AMP), cocaine, heroin, and cannabis are widely abused globally, impacting communities and increasing the risk of overdose and death. In the US, illicit drug use among college students has steadily risen from 14% in 1998 to 18% in 2017, with a peak of 21% in 2014.^1^ Even in regions with strict drug control such as China, illicit drugs and their metabolites are detected in sewage,^2^ demonstrating the global reach of this issue. These drugs not only pollute the environment but also affect the activities of organisms in nature,^3^ potentially entering the food chain and posing a threat to public health.^4^

Wastewater-based epidemiology (WBE) has evolved into a widely acknowledged interdisciplinary approach for assessing population-level health and behaviors. Over time, advancements in analytical chemistry, particularly mass spectrometry and chromatography (both gas and liquid chromatography), have enabled more precise quantification of target analytes in wastewater. Today, WBE encompasses a broad range of applications, from viral monitoring (notably used during COVID-19)^5–8^ to evaluating prescription medication use.^9^ In terms of illicit drug regulation, WBE offers near real-time insights into consumption trends and can detect surges in new psychoactive substances.^9–16^ However, current methodologies often demand centralized laboratory analyses and skilled personnel, limiting near-instantaneous response. Furthermore, challenges remain around degradation of certain drugs or metabolites in sewage, and the difficulty of accurately translating wastewater concentrations into consumption rates.^17^ Thus, while WBE remains invaluable for uncovering community drug-use trends, it still faces challenges such as high analytical costs, limited sampling frequency, and the complexity of interpreting temporally and spatially variable wastewater data. In this context, biosensors offer a unique advantage by enabling low-cost, decentralized, and high-frequency detection of specific drug biomarkers, thereby enhancing temporal resolution and supporting near real-time monitoring in the field.

Biosensors, broadly defined as analytical devices that couple a biological recognition element to a transducer,^18^ originated from the convergence of biochemistry and electronic engineering. A typical biosensor is mainly composed of three elements: (i) biological recognition elements (bioreceptor): this component is responsible for selectively binding the target molecule, (ii) transducers: physical or chemical interface that converts the biological binding event (e.g., a conformational change, catalytic reaction, or molecular interaction) into a measurable electrical, optical, or mass signal, (iii) signal processing system (processor): this element converts signal into readable form. Early milestones included enzyme-based sensors for glucose monitoring, laying the groundwork for what would become a diverse and expanding field.^19^ Over the decades, biosensors evolved in tandem with improvements in molecular biology, nanomaterials, and microfabrication, resulting in heightened sensitivity, specificity, and portability. Nowadays, biological recognition elements used in illicit drug detection include antibodies,^20–22^ enzymes,^18,19,23^ and aptamers,^24–29^ each tailored to bind specific psychoactive compounds or their metabolites. For illicit drug or other psychoactive substances monitoring, biosensors offer rapid, often label-free analyses of target molecules or metabolites, enabling a transition away from resource-heavy laboratory methods toward potentially on-site or real-time screening. Modern biosensors can detect trace levels of drugs with high selectivity, even in complex matrices such as wastewater. Their potential integration into WBE workflows could thus revolutionize how communities monitor emerging drug trends, providing near-instant insights and supporting more agile public health interventions (see Figure 1).

**Figure 1:**
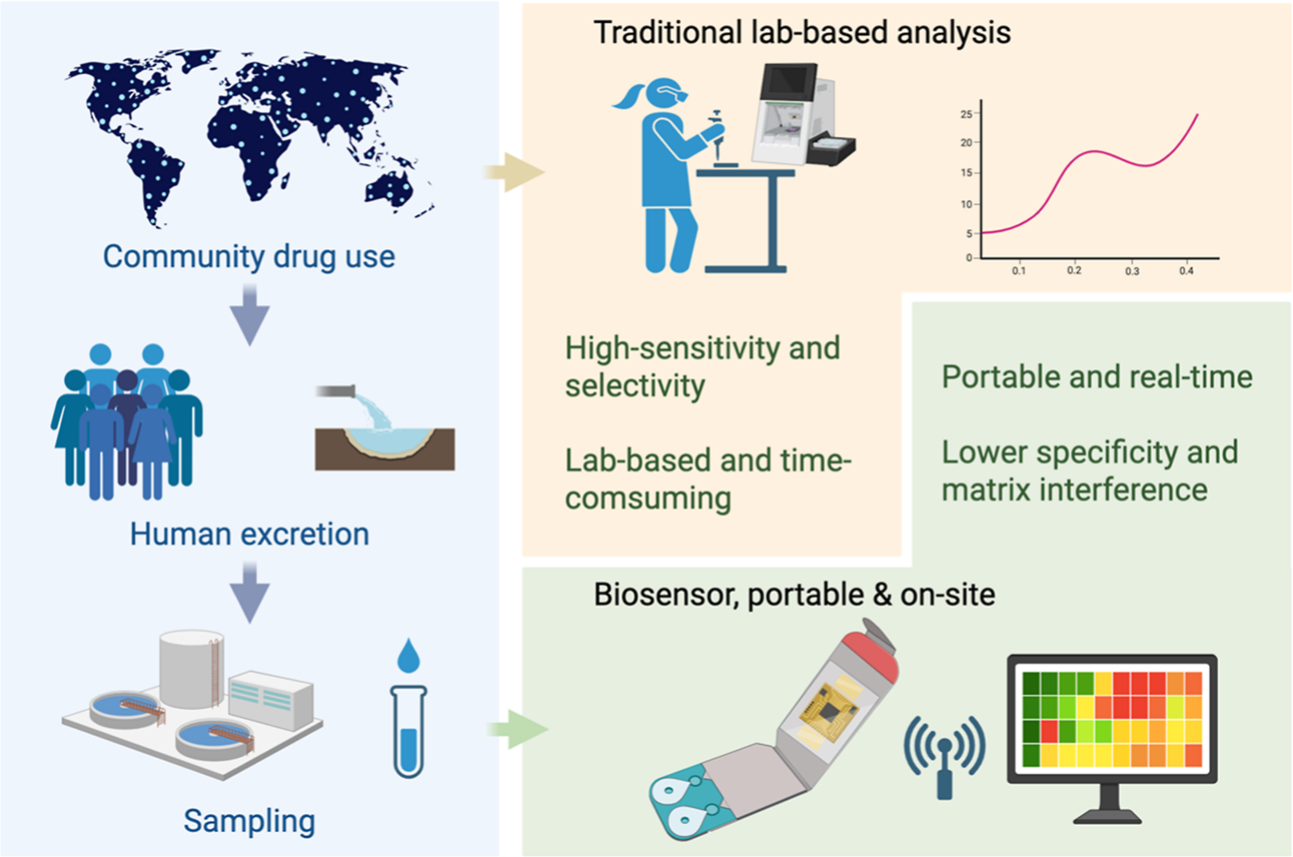
Overview of WBE workflow and comparison between traditional lab-based analysis and biosensor-enabled on-site detection for illicit drug monitoring.

This review introduces biosensor technology for WBE, emphasizing its promise for high sensitivity, selectivity, and the potential for on-site or near real-time screening of illicit drugs. By combining WBE’s broad population coverage with biosensors’ fast, cost-efficient measurements, the article explores pathways toward a more agile approach to illicit drug monitoring, detailing challenges such as matrix interference, sensor fouling, and signal stability. This review also assesses the feasibility and future outlook for integrating biosensors into WBE workflows, setting forth a vision wherein rapid detection methods align with the field’s increasing need for timely, actionable insights in both public health interventions and environmental management.

## 2 Conventional Illicit Drug Detection Techniques

Traditional lab-based analytical methods have long been used to detect and quantify illicit drugs in various matrices (e.g., biological samples, wastewater). Although these techniques are widely recognized for their high accuracy and reliability, they often require sophisticated laboratory infrastructure and extended turnaround times.

### 2.1 Chromatography–Mass Spectrometry

#### 2.1.1 Gas Chromatography–Mass Spectrometry

Gas chromatography–mass spectrometry (GC–MS) is an analytical technique that combines the features of gas chromatography and mass spectrometry to identify and quantify different substances within in complex samples. By comparing these spectra to reference standards and using retention time matching, analysts can reliably identify and quantify illicit drugs and their metabolites.^30,31^ The sensitivity and detection limits of GC–MS are highly dependent on the analyte properties and sample matrix. For instance, while some studies report limits of detection (LOD) as low as 0.06 ng L^–1^ for certain illicit drugs in artificial samples,^31^ real-world applications often require careful method optimization and validation.

Birk et al. conducted a study of the semisynthetic cannabinoid hexahydrocannabinol in seized samples from Scottish prisons, all analytes (cannabidiol, cannabinol, Δ^9^-tetrahydrocannabinol, and Δ^8^-tetrahydrocannabinol) exhibited a lower limit of quantitation (LLOQ) of 400 ng L^–1^.^32^ Langa et al.’s study on GC-MS-based analysis of amphetamine-like substances and synthetic cathinones in Portuguese wastewater influents reported a LODs of 5.08–9.63 ng L^–1^ and an LOQ of 15 ng L^–1^.^33^

While GC–MS remains a gold standard for sensitive and reliable drug identification, several practical constraints limit its utility for WBE’s emerging needs. A critical prerequisite for GC–MS analysis is that the sample must be in a form suitable for injection into the gas chromatograph. For example, GC–MS requires analytes to be sufficiently volatile or thermally stable for gas-phase separation, often necessitating chemical derivatization (e.g., silylation) that adds complexity and time. However, raw aqueous samples (e.g., wastewater) cannot be directly injected into GC–MS without extensive pretreatment (e.g., extraction, drying, derivatization), which limits its suitability for rapid, on-site monitoring. In a surveillance context that demands rapid results, this prerequisite means GC–MS cannot easily provide immediate on-site measurements. Moreover, the instrumentation is expensive, demands specialized technicians, and occupies considerable laboratory space, making GC–MS less suitable for routine on-site or field-based detection. These constraints highlight unmet needs in WBE—namely, the need for rapid, field-deployable detection methods. This gap creates space for biosensor integration, as biosensors are being designed specifically to deliver quick, on-site analysis without the infrastructure burden of GC–MS.^34,35^

#### 2.1.2 Liquid Chromatography–Mass Spectrometry

Liquid chromatography–mass spectrometry (LC–MS) is also an important technique for detecting and quantifying illicit drugs, particularly suited to nonvolatile or thermally labile compounds that resist GC–MS analysis. It offers high sensitivity, selectivity, and the ability to simultaneously quantify multiple target compounds and their metabolites in complex wastewater matrices, with limits of detection reaching the ng L^–1^ level.^34^ Nevertheless, it remains an expensive, lab-bound process requiring specialized operators, and instrument maintenance, which can limit its practicality in high-throughput or real-time monitoring scenarios.

Recent studies have demonstrated that LC–MS/MS can reliably detect a wide range of commonly abused substances, including methamphetamine, MDMA, and ketamine, especially when combined with sample pretreatment methods such as solid-phase extraction (SPE) or magnetic SPE to enhance recovery and reduce matrix interference.^14,34,35^ Liu et al. developed a DES/ZIF-MGO-based MSPE-UPLC-MS/MS method that achieved LODs as low as 0.02 μg/L with over 90% recovery in WWTP influents.^35^ Lin et al. (2024a, b) implemented high-resolution HPLC–MS/MS protocols across both WWTP and sewer-network samples in Taiwan. By back-calculating from the measured concentrations, they estimated community drug consumption, reporting usage levels of methamphetamine and ketamine in the range of 22–740 mg/day/1000 people.^14,34^

LC–MS extends detection to a broader range of drug compounds (including those not amenable to GC–MS) with high sensitivity and selectivity, but its operational demands similarly underscore a gap in the WBE. In practice, traditional LC–MS workflows involve extensive sample preparation, such as filtration, solid-phase extraction (SPE), or derivatization, that can add anywhere from 30 min to several hours of upfront work, especially for complex matrices like wastewater. For a single LC/GC–MS run once the sample is ready, 15–40 min is a typical analytical window.^36^ For example, a study developed a fast LC–MS/MS screening method that detects 739 bioactive compounds in blood and urine within just 18 min per run, using minimal sample preparation.^37^

Recent streamlining efforts (e.g., direct injection (DI) protocols that reduce preparation time) have not overcome the fundamental lack of portability.^38,39^ The capability of direct-injection LC–MS/MS for high-throughput analysis of complex environmental matrices has been successfully demonstrated in large-scale monitoring campaigns. As evidenced by a comprehensive year-long study in Ireland, a DI-LC-MS/MS method enabled the rapid quantification of over 100 contaminants of emerging concern (CECs), including pharmaceuticals, pesticides, and personal care products, in wastewater influent, effluent, and receiving waters at subng/L levels.^40^ Nevertheless, a study employing a 5.5 min chromatographic method requiring only 10 μL of filtered sample successfully quantified 102 contaminants of emerging concern (CECs) at low ng/L levels across six rivers in Germany and Switzerland. This high-throughput approach enabled the collection of over 260 injections per day, facilitating a detailed mapping of CEC sources (e.g., WWTP outfalls) and their rapid dilution downstream over short distances.^41^ These approaches significantly reduced sample preparation time and complexity while maintaining robust performance, providing a valuable tool for acquiring the extensive temporal and spatial data necessary for environmental risk assessment and prioritization.

In summary, GC/LC–MS and biosensors present a clear trade-off between analytical performance and practical applicability in illicit drug monitoring. GC/LC–MS is the undisputed reference technique for confirmatory analysis, offering exceptional sensitivity, selectivity, and the ability to simultaneously quantify a broad spectrum of drugs and metabolites at trace (ng/L) levels, even in complex matrices like wastewater. However, these capabilities come at the cost of high expense, lack of portability, and the need for specialized operators, rendering it impractical for real-time, on-site decision-making. Biosensors, in contrast, address the critical gaps of portability, real-time, and cost-effectiveness, enabling rapid, decentralized screening. Their limitations typically lie in lower multianalyte capability, potentially higher susceptibility to matrix effects, and generally higher limits of detection compared to GC/LC–MS. Thus, while GC/LC–MS remains essential for definitive laboratory-based quantification and large-scale retrospective studies, biosensors offer a complementary technology for high-throughput, rapid and on-site screening and early warning systems.

### 2.2 Raman Spectroscopy and Surface-Enhanced Raman Spectroscopy

Raman spectroscopy relies on inelastic scattering of monochromatic light, typically from a laser, to probe the vibrational modes of molecules. When photons interact with the molecular bonds, most scatter elastically (Rayleigh scattering) at the same wavelength, but a small fraction undergo inelastic scattering, referred to as the Raman effect, resulting in photons that shift in energy (and thus wavelength) relative to the incident light. Each molecule exhibits characteristic Raman shifts corresponding to specific vibrational transitions, creating a fingerprint that can be used to identify and characterize the compound.

Surface-enhanced Raman spectroscopy (SERS) is an advanced analytical technique that significantly amplifies Raman signals by exploiting localized surface plasmon resonance (LSPR) on metallic nanostructures, typically gold or silver nanoparticles, or Au/Ag-based nanostructures. This enhancement occurs due to the intense electromagnetic field generated near the nanoparticle surface when incident light interacts with the conduction electrons in the metal, creating localized surface plasmons. Molecules adsorbed onto or in close proximity to these metal surfaces experience dramatically enhanced Raman scattering, improving detection sensitivity by several orders of magnitude from 10^6^ to 10^14^ as reported.^17,42^

Raman enhancement substrate, especially nanoparticles, play a crucial role in SERS performance. Gold and silver nanoparticles are commonly employed due to their strong plasmonic properties in the visible and near-infrared spectral ranges. Additionally, core–shell nanoparticles (e.g., Au@Ag)^43,44^ further improve performance by combining the chemical stability of gold with the stronger electromagnetic enhancement of silver. Factors such as nanoparticle size, shape, and surface chemistry are critical in maximizing enhancement effects.

In the context of illicit drug detection, the ability of Raman spectroscopy to produce detailed vibrational profiles with minimal sample preparation makes it an attractive alternative to more labor-intensive techniques such as GC/LC–MS.^45,46^ Despite its powerful detection capabilities, Raman spectroscopy faces challenges when applied in real-world samples such as wastewater. These include background interference from fluorescent contaminants, matrix complexity, and variability in nanoparticle aggregation. Consequently, combining SERS with effective sample preparation methods, such as solid-phase extraction or filtration, is often necessary to achieve optimal results in environmental monitoring scenarios.

### 2.3 Colorimetric Tests

Colorimetric tests are rapid chemical assays that produce characteristic color changes upon reaction with specific drug classes.^47^ Common examples include the Marquis test (often used to screen for amphetamines and certain opioids), the Scott test (for cocaine), and various reagent kits tailored to particular substance groups. These assays are popular in preliminary field operations or law enforcement settings due to their portability, ease of use, and low cost, typically requiring only a small sample and a few drops of reagent. However, spot tests are inherently qualitative or semiquantitative: the resulting color shift indicates the likely presence (or class) of a drug but usually cannot confirm identity with precision. They are also prone to false positives or cross-reactivity with chemically similar compounds. Despite these limitations, colorimetric spot tests remain an essential first-line screening tool, enabling quick, on-site decisions before more rigorous laboratory analysis.

## 3 Biosensors for Illicit Drug Detection

Biosensors for illicit drug detection offer a promising alternative to traditional analytical techniques, providing rapid, sensitive, and potentially field-deployable solutions for identifying drugs and their metabolites. Biosensors have demonstrated excellent capability in detecting illicit drugs in multiple matrices, reported to achieve detection limits comparable to those of traditional laboratory methods.^28,48–53^ Depending on the detection mechanism, biosensors can be classified into various types, with electrochemical and optical biosensors being the most widely explored for illicit drug monitoring. Electrochemical biosensors measure changes in electrical properties (such as current, voltage, or impedance) upon drug binding, offering high sensitivity in complex environments like wastewater or biological fluids. Optical biosensors, on the other hand, rely on light-based interactions (such as fluorescence, surface plasmon resonance, or Raman scattering) to provide label-free, highly specific drug detection. Given their potential for real-time monitoring, miniaturization, and low-cost manufacturing, biosensors are increasingly being investigated for applications ranging from forensic drug screening to WBE. Figure 2 shows biosensor-based detection of illicit drugs in wastewater.

**Figure 2:**
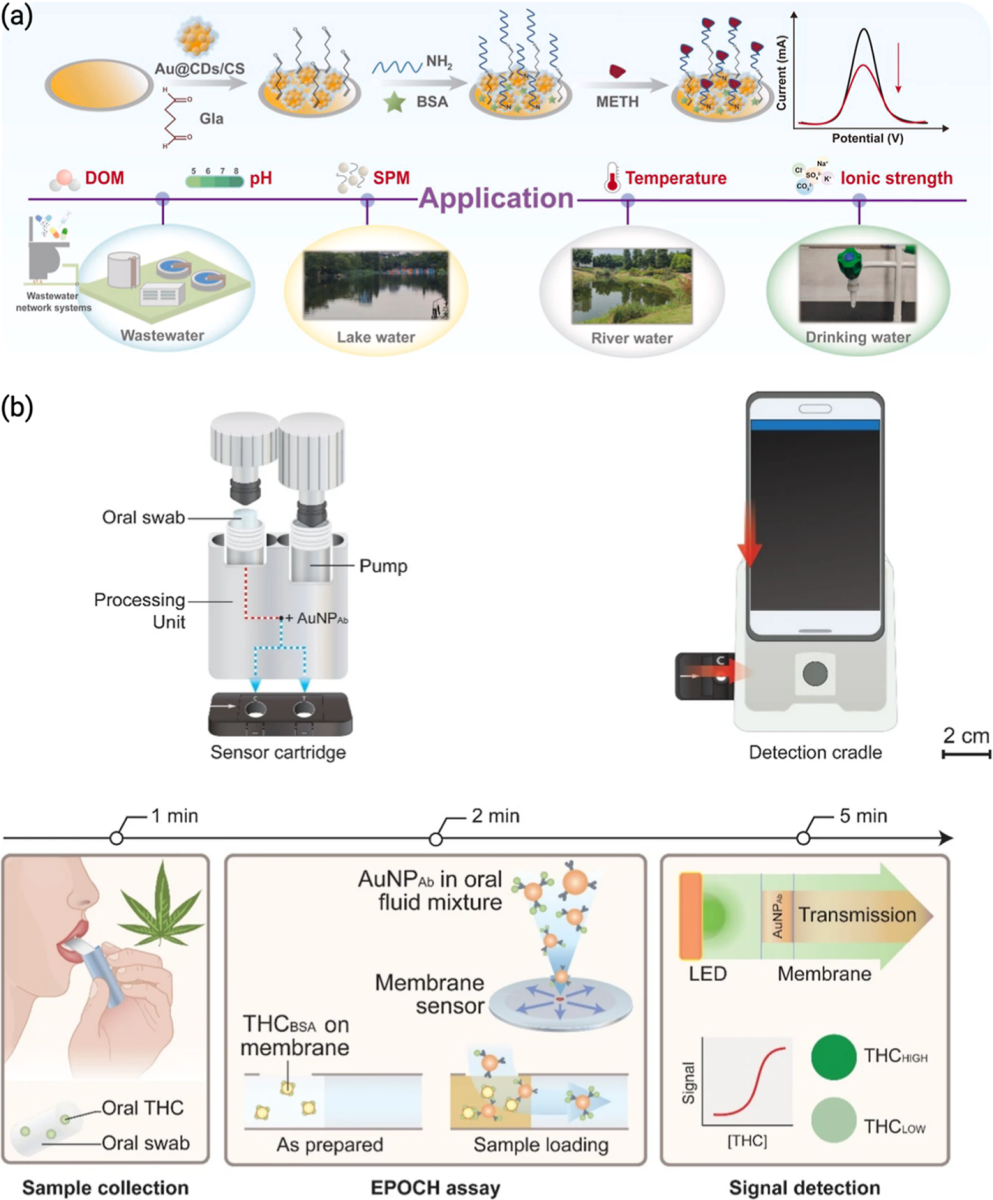
(a) Illustration of ultrasensitive detection of methamphetamine in environmental water bodies by an Au@carbon dots/chitosan nanocomposite modified electrochemical aptasensor.^54^ Reproduced with permission from ref 54. Copyright 2025 Elsevier. (b) Illustration of the detection of THC in saliva using an express probe for on-site cannabis inhalation.^55^ Reproduced from ref 55 under the Creative Commons CC-BY license. Copyright 2024 Springer.

### 3.1 Biological Recognition Elements

Biosensors rely on highly sensitive and selective biological recognition elements to detect illicit drugs and their metabolites in complex matrices such as sewage.^56^ The core function of a biological recognition element in a biosensor is to selectively bind to the target drug molecule and generate a detectable signal. The working principle of each recognition element depends on the nature of the binding interaction and how it is transduced into a measurable response.

An ideal biological recognition element should fulfill several essential criteria to ensure reliable performance in sensor systems. First, high selectivity is crucial that the element must be able to distinguish the target analyte from structurally similar compounds; for example, aptamers have been engineered to differentiate cocaine from its analogs with high specificity.^56^ Second, chemical and thermal stability is important to maintain activity under varying environmental conditions such as changes in pH, temperature, or ionic strength—this is particularly critical for field-deployable sensors used in wastewater or environmental samples. Third, the recognition element must exhibit strong and rapid binding affinity to the target, as seen in antibody–antigen interactions which typically display nanomolar dissociation constants and fast association kinetics.^20^ Fourth, compatibility with the sensor platform is essential: for instance, thiol-modified aptamers can be readily immobilized on gold nanoparticles or electrodes without compromising their recognition ability. Lastly, reproducibility and scalability are vital for practical applications; synthetic elements like molecularly imprinted polymers (MIPs) offer excellent batch-to-batch consistency and can be mass-produced more easily than biological materials such as monoclonal antibodies.^57^

#### 3.1.1 Enzymes

Enzymes catalyze specific biochemical reactions that involve the target drug or its metabolites, producing a detectable byproduct (e.g., electrons, protons, or colored compounds).^58^ For example, glucose oxidase (GOx) specifically catalyzes the oxidation of glucose into gluconic acid, producing hydrogen peroxide as a byproduct, which can be electrochemically detected.^19,59^ Enzymes offer several advantages, including high reaction rates, excellent specificity, and compatibility with a wide range of analytes. However, they also present certain limitations. Enzymes are often sensitive to environmental conditions such as temperature, pH, and the presence of organic solvents, which may lead to denaturation or loss of activity.^18,23,60^ They can also be inhibited by interfering substances commonly found in real samples. To overcome these drawbacks, various strategies have been developed. Enzyme immobilization techniques help stabilize enzymes by anchoring them onto nanomaterials, electrodes, or hydrogels, enhancing their operational stability and reusability. Protein engineering and directed evolution approaches have been applied to improve enzyme stability and resistance to inhibitors.^18,23^ Additionally, hybrid systems combining enzymes with nanomaterials like graphene oxide or metal–organic frameworks (MOFs) have shown promise in enhancing catalytic performance, achieving a detection limit as low as 0.5 nM and a wide linear range of 8 to 500 nM for MAMP detection.^61^ Modern enzyme-based biosensor research focuses on enzyme-nanoparticle hybrids and multienzyme cascade reactions to improve specificity and detection limits, but enzyme biosensors still require optimized storage and handling to maintain long-term performance.

#### 3.1.2 Antibodies

Antibodies are widely used in immunosensors due to their highly specific antigen–antibody interactions, which allow selective recognition of illicit drugs and their metabolites. These biosensors typically rely on electrochemical, fluorescence, or surface plasmon resonance (SPR) transduction, where drug binding triggers a detectable signal. Immunosensors have been successfully applied in portable drug screening devices for substances such as cocaine, THC, methamphetamine, and fentanyl, with reported detection limits in the picomolar (pM) range.^45,62,63^

The main advantages of antibodies include exceptional specificity, strong binding affinity, and the ability to detect low-abundance targets in complex matrices like wastewater. However, antibodies can be sensitive to environmental conditions such as temperature, pH, and ionic strength, potentially leading to denaturation or reduced binding efficiency.^20^ Moreover, their production involves biological systems (e.g., animals or cell cultures), which can be costly and time-consuming.

To improve antibody performance in biosensors, various strategies have been explored. Immobilization on stable substrates like gold nanoparticles or carbon-based materials enhances their stability and preserves binding activity. Genetic engineering has enabled the development of recombinant antibodies and fragments (e.g., single-chain variable fragments, scFvs) with improved thermal stability and reduced size for better sensor integration. Additionally, synthetic alternatives such as aptamers and molecularly imprinted polymers (MIPs) are being explored to overcome the limitations of traditional antibodies, especially for use in field-deployable, real-time biosensing systems.

#### 3.1.3 Molecularly Imprinted Polymers

Molecularly imprinted polymers (MIPs) serve as synthetic receptors that mimic the binding sites of biological molecules, offering a low-cost, highly stable alternative for illicit drug detection. MIPs are fabricated by polymerizing monomers around a template drug molecule, leaving behind specific molecular cavities after template removal that selectively rebind the target drug. These synthetic recognition elements are particularly attractive for cocaine, MDMA, and THC detection, as they resist temperature, pH variations, and enzymatic degradation, making them ideal for environmental and forensic applications. However, MIPs generally have lower binding affinity than biological receptors, require precise template removal processes to prevent nonspecific binding, and may exhibit reduced selectivity in complex samples like wastewater or blood. In the literature analyzed in this study regarding biosensors for illicit drug detection, MIP-based biosensors generally exhibit higher LOD and perform poorly in complex matrices such as wastewater.^50,57,64–66^ This is because: (i) MIPs typically have lower binding affinity than biological receptors due to their synthetic nature; (ii) MIPs suffer from nonspecific binding in complex sample matrices like wastewater, where multiple organic and inorganic contaminants can interfere with detection; (iii) MIP-based biosensors often have slower binding kinetics and require longer incubation times compared to antibody- or aptamer-based biosensors.^67^ Recent advancements focus on nanoMIPs (nanoscale imprinted polymers) and electropolymerized MIPs, which enhance binding efficiency and signal transduction, paving the way for more reliable real-time illicit drug monitoring in portable sensing platforms.

#### 3.1.4 Aptamers

Aptamers are single-stranded DNA or RNA molecules selected through the systematic evolution of ligands by exponential enrichment (SELEX) process, offering a synthetic alternative to antibodies for drug biosensing.^67^ Aptamers bind to target drugs through their unique three-dimensional folding structures, with high specificity and better chemical stability than antibodies. They can be integrated into electrochemical, fluorescence, and surface plasmon resonance (SPR) biosensors, enabling highly sensitive detection of drugs such as cocaine, 3,4-methylenedioxymethamphetamine (MDMA), and synthetic opioids, often achieving nanomolar (nM) or lower detection limits.^24,26,54,68–74^

Several studies have demonstrated the application of aptamer biosensors in detecting illicit drugs within wastewater matrices. For instance, an electrochemical aptasensor achieved an LOD of 0.87 pg L^–1^ for methamphetamine in real water samples (drinking water, river, lake, and wastewater), showing excellent recovery (92.4–104.6%) and stability across common environmental conditions, supporting its applicability in WBE.^54^ Similarly, a fluorescent aptasensor based on UiO-66/AuNPs nanocomposites enabled cocaine detection in human serum with a detection limit of 0.178 μM and high selectivity, demonstrating applicability in real biological matrices.^75^

Despite these advantages, aptamers face challenges such as lower binding affinity than antibodies, potential structural degradation in biological fluids, and the labor-intensive SELEX process required for aptamer selection. Ongoing advancements include modified aptamers with chemical stabilization and aptamer-nanoparticle conjugates to enhance signal amplification and improve real-world applicability in drug monitoring.

In addition to current aptamer-, antibody-, and MIP-based biosensors, emerging biosensor technologies are being developed with promising potential for wastewater-based drug monitoring. For instance, wearable biosensors, incorporating flexible electrochemical or optical modules, have also been proposed for decentralized environmental surveillance.^76,77^ Moreover, microfluidic-integrated biosensors enable automated, multiplexed analysis with minimal sample consumption, ideal for field-deployable systems.^17,78,79^ These alternative biosensor platforms, though not yet widely applied in WBE, offer scalable, real-time, and cost-effective solutions for future illicit drug monitoring in aquatic environments.

### 3.2 Apparent Binding Affinity (*K*_d_)

The binding affinity of biological recognition elements is a key determinant of biosensor sensitivity, often characterized by the dissociation constant (*K*_d_). The *K*_d_ values between biosensor recognition elements and target analytes can be significantly altered in complex wastewater environments compared to ideal buffer conditions. For monoclonal antibodies, *K*_d_ values typically range from 10^–9^ to 10^–12^ M, indicating high affinity toward their target antigens.^20,80^ Aptamers usually exhibit *K*_d_ values in the low nanomolar to midpicomolar range (10^–9^ to 10^–12^ M), comparable to antibodies, though more variable depending on target type and sequence optimization.^28,29^ Enzymes demonstrate substrate affinities in the micromolar to nanomolar range (10^–6^ to 10^–9^ M) depending on their catalytic turnover rate and Michaelis–Menten constant (Km).^18,23^ In contrast, MIPs generally display weaker binding affinities, with reported *K*_d_ values ranging from 10^–6^ to 10^–3^ M, due to their synthetic nature and less specific interaction mechanisms.^57,65,66,81^ Figure 3 shows the four biological recognition elements discussed in Section 3.1 and the reported *K*_d_ ranges of the four biological recognition elements.

**Figure 3:**
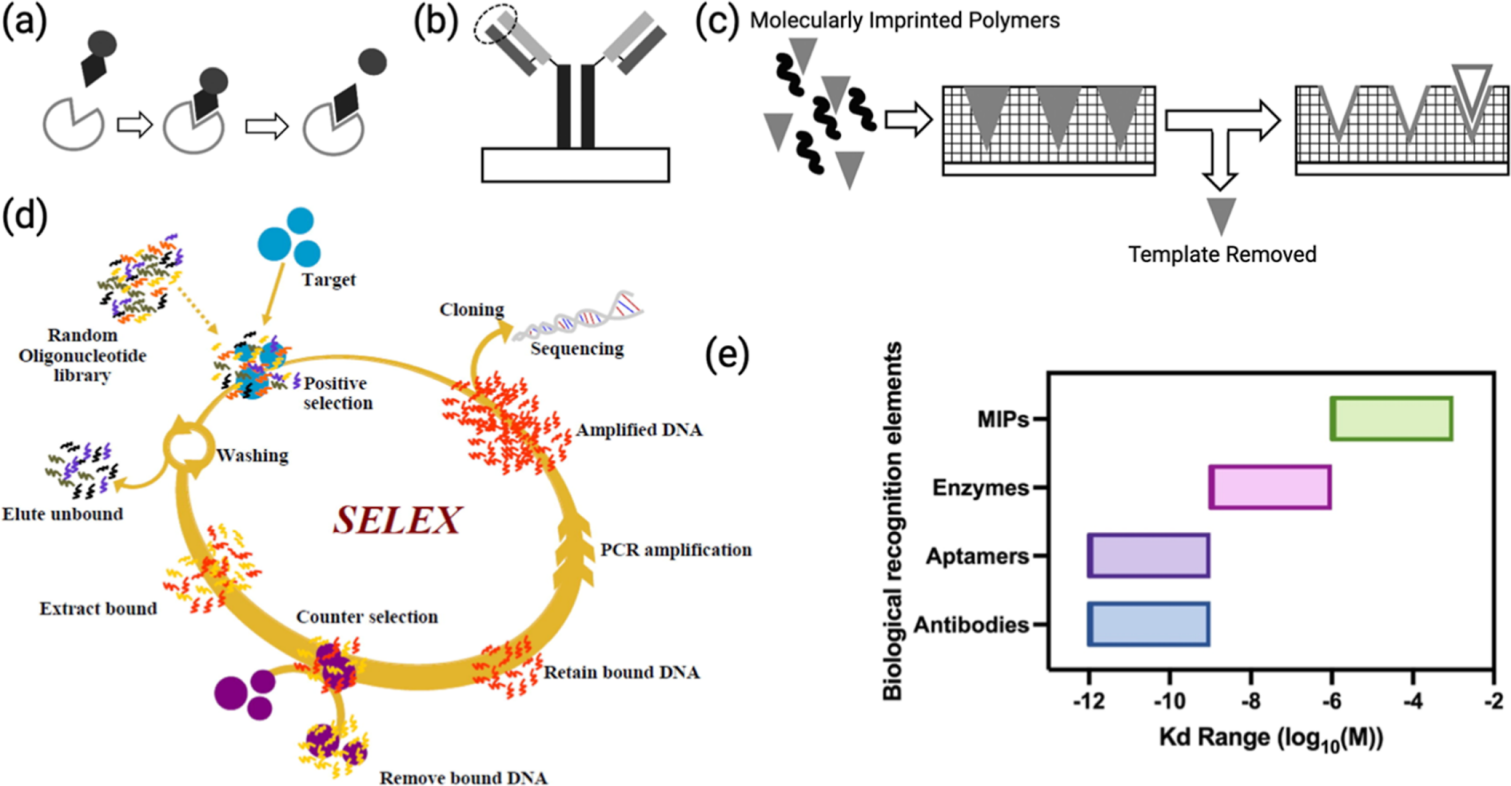
Illustration of four commonly used biological recognition elements. (a) Enzymes catalyze specific biochemical reactions that involve the target, producing a detectable byproduct.^67^ Reproduced with permission from ref 67. Copyright 2018 American Chemical Society. (b) 3D confirmation of antibodies is “Y” shaped with binding domains, circled above, typically located on the distal end.^67^ Reproduced with permission from ref 67. Copyright 2018 American Chemical Society. (c) Synthetic polymerization encapsulating the target bioanalyte forms an analyte binding site.^67^ Reproduced with permission from ref 67. Copyright 2018 American Chemical Society. (d) Aptamers selected through the SELEX process.^73^ Reproduced with permission from ref 73. Copyright 2022 Elsevier. (e) Comparison of *K*_n_ ranges for common biological recognition elements.

Wastewater contains a diverse array of interfering substances, such as organic matter, surfactants, heavy metals, and suspended particulates, that can interfere with the molecular recognition process. Fluctuations in pH and ionic strength can induce conformational changes in aptamers or antibodies, disrupting the integrity of their binding sites and reducing their affinity for the target molecule. Additionally, high salt concentrations may shield electrostatic interactions essential for binding, while organic contaminants or colloids may nonspecifically adsorb to the sensor surface, sterically hindering target access. These matrix effects often result in an increased apparent *K*_d_, effectively lowering sensor sensitivity and reliability. Therefore, biosensor designs for WBE must carefully account for matrix-induced affinity shifts. For example, aptamer-based cocaine sensors have shown an increase in apparent *K*_d_ from 1.2 nM in buffer to over 20 nM in raw influent wastewater due to ionic strength and organic fouling.^28^ To mitigate these effects, antifouling strategies such as PEGylated surfaces, zwitterionic polymer brushes, or nanocomposite coatings (e.g., graphene-oxide-PEG) have been employed to reduce nonspecific adsorption by up to 80% in simulated wastewater.^64^ Furthermore, chemically stabilized recognition elements like thioether-modified aptamers and recombinant single-chain antibodies have demonstrated improved binding fidelity in pH-variable samples.^82^ Real-world calibration is often achieved via matrix-matched standard curves or standard addition methods, ensuring that detection sensitivity and selectivity remain consistent when translating from lab to field conditions.

### 3.3 Electrochemical Biosensor for Illict Drug Detection

Electrochemical biosensors operate by converting biochemical interactions between a target drug molecule and a biological recognition element into electrical signals (such as current, voltage, or impedance). Given their analytical performance and compatibility with low-cost, decentralized monitoring platforms, electrochemical biosensors are poised to become a key enabling technology for WBE applications, potentially replacing traditional approaches in certain contexts. To achieve the sensitivity and robustness required for complex wastewater matrices, these sensors often incorporate signal amplification strategies to enhance the measurable electrical response (e.g., current, voltage, impedance) resulting from the target-recognition event, thereby lowering the detection limit and improving reliability under field conditions.

One of the most widely used approaches is enzyme-mediated amplification, where catalytic reactions generate detectable electrochemical signals. For instance, in systems using glucose oxidase (GOx) or horseradish peroxidase (HRP), target binding triggers redox reactions that produce hydrogen peroxide or reduce electroactive substrates such as TMB. Yang et al. demonstrated that a dual-enzyme cascade system using GOx and HRP on an AuNP-modified immunoelectrode improved the detection limit of methamphetamine in serum from 1 ng L^–1^ to 0.001 μg L^–1^, representing a 1000-fold enhancement compared to unmodified sensors.^80^

Nanomaterial-assisted amplification is another critical strategy. Conductive nanomaterials such as gold nanoparticles (AuNPs), carbon nanotubes (CNTs), and graphene oxide (GO) increase the effective electrode surface area and facilitate electron transfer. For example, De Rycke et al. showed that capacitive sensors functionalized with AuNPs exhibited a 3.7-fold improvement in sensitivity for amphetamine detection in wastewater compared to flat electrodes.^66^ El-Akaad et al. developed a capacitive MIP-Au biosensor for detecting 4-methyl-5-phenylpyrimidine (4M5PP), a forensic marker of illicit ATS synthesis (Figure 4a). The sensor showed a linear range of 17.0–510.6 μg/mL with a detection limit of 13.6 μg/mL and achieved 95–101% recovery in real wastewater samples, maintaining over 90% signal after 24 reuse cycles. This study demonstrates a robust strategy for indirect environmental monitoring of clandestine drug production.^57^ Sengel et al. developed an electrochemical immunosensor based on an electroactive peptide-modified electrode (EDOT-BTDA-PPhe) for the detection of cocaine and its major metabolite, benzoylecgonine (BE) (Figure 4b). The sensor exhibited a linear response between 151.7 and 7584 μg/L with a detection limit of 124.4 μg/L (*R* ^2^ = 0.998), and achieved recovery rates between 91.9% and 98.7% in spiked urine and saliva samples.^83^ Oueslati et al. developed a low-cost capacitive aptasensor enhanced by AC electroosmotic flow for the on-site detection of cocaine (Figure 4c). The sensor achieved an ultralow detection limit of 2.37 ng/L in buffer and 4.06 ng/L in serum within 30 s, demonstrating a wide linear range (4.4–4.4 ng/L) and excellent selectivity against structurally similar molecules.^84^

**Figure 4:**
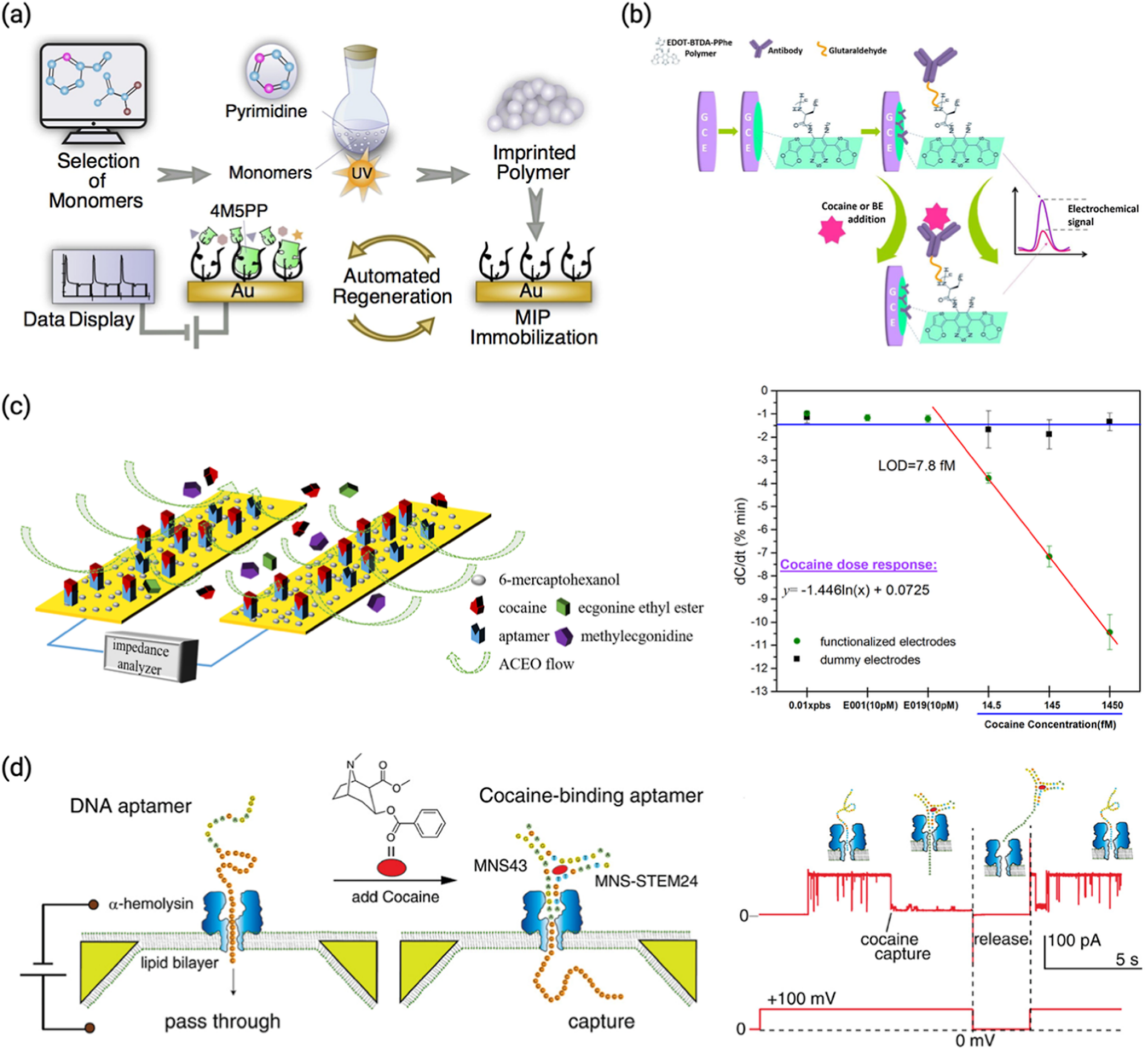
(a) Schematic illustration of the preparation of the 4M5PP-MIP-Au biosensor.^57^ Reproduced with permission from ref 57. Copyright 2021 Elsevier. (b) Surface modification and the measurement principle of the EDOT-BTDA-PPhe-antibody biosensor.^83^ Reproduced with permission from ref 83. Copyright 2017 Elsevier. (c) Schematic illustration of an aptamer-based electrochemical sensor enhanced by ACEO-induced flow for cocaine detection. The ACEO effect facilitates the transport of cocaine molecules toward the functionalized electrode, improving binding efficiency and response speed. Response of cocaine in 0.01 × PBS on both functionalized sensors and dummy sensors.^84^ Reproduced with permission from ref 84. Copyright 2018 Elsevier. (d) Schematic overview of detection using the cocaine-binding aptamer (left). The DNA aptamer can pass through the pore in the absence of cocaine. However, in the presence of cocaine, the CBA cannot pass through and is captured in the pore. Typical current–time traces for DNA aptamers with cocaine in 1.0 M KCl, 10 mM PBS, and 1 mM EDTA (pH 7.4).^85^ Reproduced with permission from ref 85. Copyright 2011 American Chemical Society.

In addition, electroactive labels such as ferrocene or methylene blue are widely used to improve output signal strength. In a study by Oueslati et al., ferrocene-labeled aptamer probes for cocaine detection demonstrated a 100-fold increase in signal-to-background ratio when conjugated to AuNPs, reducing the LOD to 2.4 × 10^–9^ μg L^–1^ in serum samples.^84^ These strategies are often integrated; for example, aptamer-AuNP-ferrocene constructs combine recognition, signal transduction, and amplification into a single compact system capable of subnanomolar drug detection even under variable matrix conditions. Table 1 summarizes the recent developments in electrochemical biosensor for illicit drug detection in wastewater or biological fluids.

**Table 1:** Electrochemical Biosensors for Illicit Drug Detection in Wastewater and Biological Fluids^a^

sensors	analytical method	sample matrix	target	LOD (μg L^–1^)	linear range (μg L^–1^)	refs
MIP-Au	capacitive	wastewater	AMP	∼1.00 × 10^4^	1.35 × 10^4^–4.06 × 10^5^	^57^
MIP-Au	capacitive	wastewater	AMP	∼4.13 × 10^3^	NR	^66^
			NFA	∼3.75 × 10^3^	NR	
			BMK	∼6.76 × 10^3^	NR	
MIP-based SPCE	SWV	blood, urine	MDMA	1.53 × 10^2^	4.83 × 10^2^–3.86 × 10^4^	^50^
antibody-Au	EIS	serum	MAMP	1.00 × 10^–3^	2.00 × 10^–3^–2.00 × 10^–1^	^80^
			morphine	3.00 × 10^–4^	4.00 × 10^–3^–8.00 × 10^–2^	
EDOT-BTDA-PPhe-antibody	EIS	saliva, urine, and human serum	cocaine and BE	∼1.20 × 10^2^	1.52 × 10^2^–7.58 × 10^3^	^83^
GPH-SPE/PdNP-MIPs	SWV	river water and saliva	cocaine	∼1.50 × 10^4^	3.00 × 10^4^–1.50 × 10^5^	^49^
aptamer-Au	ICS	serum	cocaine	2.37 × 10^–9^	4.40 × 10^–9^–4.40 × 10^–3^	^84^
DNA aptamer	DPV		codeine	1.71 × 10^–3^	2.19 × 10^–3^–2.19	^86^
aptamer	CV	serum	cocaine	1.00 × 10^1^	3.00 × 10^1^–3.0 × 10^2^	^70^
aptamer	DPV	serum	Cocaine	3.00 × 10^–2^	3.00 × 10^–1^–3.33 × 10^3^	^69^
aptamer	DPV	serum	cocaine	3.00 × 10^–2^	3.00 × 10^–1^–3.33 × 10^3^	^87^
DNA	DPV	serum	ketamine	1.28 × 10^–1^	0–1.43 × 10^2^	^88^
aptamer	DPV	serum	cocaine	7.00 × 10^–2^	9.00 × 10^–2^–4.55	^68^
aptamer	DPV	synthetic biological fluids	cocaine	4.55 × 10^–1^	7.58 × 10^–1^–3.03	^89^
DNA aptamer	DPV		codeine	9.00 × 10^–4^	3.00 × 10^–3^–3.00 × 10^1^	^90^
aptamer	DPV	serum	codeine	9.00 × 10^–5^	3.00 × 10^–4^–3.00 × 10^1^	^91^

a NFA = *N*-formyl amphetamine; BMK = benzyl methyl ketone; NR = not reported; SPCE = screen printed carbon electrode; SWV = square wave voltammetry; EIS = electrochemical impedance spectroscopy; BE = benzoylecgonine; ICS = interfacial capacitance sensing; DPV = differential pulse voltammetry; CV = cyclic voltammetry.

MIP-based biosensors are featured in multiple studies, often coupled with capacitive and SWV,^50,57,66^ and are the only kind of biosensor applied in wastewater detection. However, MIP-based biosensors exhibit higher LODs, making them less suitable for trace drug detection, especially in wastewater and environmental samples where ultralow concentrations are of interest. Among all the reported electro biosensors for illicit drug detection, Yang et al. reported the lowest LOD of 0.001 μg/L for MAMP and 0.0003 μg/L for morphine with a convincing linear range to quantitatively assess the concentration of illicit drugs in complex matrix.^80^

### 3.4 Optical Biosensor for Illicit Drug Detection

Optical biosensors convert molecular interactions into optical signals, such as color changes, fluorescence, SERS, or absorbance shifts. These sensors leverage the unique interactions between drugs and biological or nanomaterial-based recognition elements to produce highly specific and sensitive detection. Compared to electrochemical biosensors, optical biosensors often exhibit higher sensitivity and multiplexing capabilities, making them particularly useful for on-site drug screening and forensic analysis.

In the context of wastewater-based epidemiology, signal amplification of optical biosensor is particularly critical due to the low abundance of analytes and high background interference. Optical biosensors utilize a wide range of amplification strategies, including nanoparticle-mediated plasmonic enhancement, catalytic color development, and resonance-based signal transduction, to improve detection performance.

Among these, colorimetric biosensors are widely adopted for their simplicity, cost-effectiveness, and compatibility with portable devices. Signal amplification in colorimetric platforms is typically achieved using the aggregation of gold or silver nanoparticles (AuNPs/AgNPs). Upon target binding, aptamer-functionalized nanoparticles undergo aggregation or dispersion, resulting in a visible color change (e.g., red-to-blue shift). Mao et al. demonstrated a duplex AuNP-based aptasensor for methamphetamine and cocaine in wastewater, achieving detection limits of 0.075 μg L^–1^ for MAMP and 1.0 μg L^–1^ for cocaine, with recovery rates above 85% in spiked wastewater samples.^28^ Notably, colorimetric sensors without plasmonic amplification generally exhibit LODs in the mg/L range, underscoring the essential role of nanoparticle-enhanced signal readouts.

Surface-enhanced Raman scattering (SERS) offers one of the most powerful amplification mechanisms in optical biosensing. By leveraging the electromagnetic field enhancement near nanostructured metal surfaces, especially AuNPs, AgNPs, and Au@Ag core–shells which have been reported to successfully enhance the sensitivity of biosensors for illicit drug detection, SERS can amplify Raman signals by factors ranging from 10^6^ to 10^14^, enabling ultratrace detection of analytes. For instance, Mao et al. reported a paper-based SERS nanosensor for methamphetamine in wastewater with a detection limit of 0.0072 μg L^–1^, covering a linear range from 0.1 to 10,000 μg L^–1^, suitable for both low-concentration surveillance and high-exposure events.^53^ Similarly, a SERS biosensor using Au@Ag core–shell nanoparticles achieved an LOD of 0.16 μg L^–1^ for MAMP in urine samples, outperforming conventional fluorescence-based immunoassays.^44^

In addition to SERS and colorimetry, fluorescence amplification strategies are also used. These include quantum dot labeling, fluorescence resonance energy transfer (FRET), and chemically stabilized fluorescent aptamers. Although their sensitivity is generally lower than SERS, fluorescent biosensors offer real-time kinetic monitoring and multiplexing. For example, Roncancio et al. developed an aptamer-fluorophore assembly for cocaine detection in serum, urine, and saliva, reaching LODs in the low μg L^–1^ range (∼3–11 mg L^–1^), though with limited matrix tolerance.^71^ Table 2 summarizes the recent developments in optical biosensor for illicit drug detection in wastewater or biological fluids.

**Table 2:** Optical Biosensors for Illicit Drug Detection in Wastewater and Biological Fluids^a^

sensor matrix	analytical method	sample matrix	target	LOD (μg L^–1^)	linear range (μg L^–1^)	refs
AuNP-based biosensor	colorimetry	wastewater	MAMP	7.50 × 10^–2^	NR	^28^
			cocaine	1.00	NR	
AgNPs on diatomaceous earth films	SERS	wastewater	fentanyl	NR	NR	^92^
paper-based nanosensor	SERS	wastewater	MAMP	7.20 × 10^–3^	1.00 × 10^–1^–1.00 × 10^4^	^51^
Au@Ag core–shell NP-based biosensor	colorimetry	urine	MAMP	2.50 × 10^–2^	7.50 × 10^–2^–3.00 × 10^1^	^93^
			cocaine	1.50 × 10^–1^	3.00 × 10^–1^–4.50 × 10^1^	
Au@Ag core–shell NP-based biosensor	SERS	urine	MAMP	1.60 × 10^–1^	5.00 × 10^–1^–4.00 × 10^1^	^44^
DNAzyme MB-based biosensor	colorimetry	urine	MAMP	7.46 × 10^1^	1.19–7.46 × 10^1^	^94^
aptamer	fluorescence	urine, saliva, serum, water	cocaine	∼5.58 × 10^3^	NR	^71^
				∼3.15 × 10^3^	NR	
				∼1.09 × 10^4^	NR	
				∼6.07 × 10^1^	NR	
fluorescent-based immunoassay	fluorescence	saliva	cannabis (THC)	2.50 × 10^1^	NR	^95^
aptamer-AuNP	colorimetry	urine	MAMP	7.50 × 10^–2^	1.50 × 10^–1^–3.00 × 10^1^	^96^
aptamer and peroxidase-mimicking DNAzyme	colorimetry	spiked biologic fluid	cocaine	1.52 × 10^3^	3.00 × 10^3^–3.00 × 10^5^	^97^
aptamer-graphene oxide	fluorescence	spiked human plasma	cocaine	3.00 × 10^–2^	3.00 × 10^–2^–1.52 × 10^2^	^98^
aptamer-SiNP	fluorescence	serum	cocaine	2.50 × 10^–2^	1.50 × 10^–1^–2.43 × 10^1^	^72^
aptamer-SiNP/AuNP	fluorescence	serum	cocaine	6.00 × 10^–2^	1.50 × 10^–1^–6.07	^99^

a AuNP = gold nanoparticle; AgNP = silver nanoparticle; DNAzyme MB = G-quadruplex–hemin DNAzyme molecular beacon; SiNP = silica nanoparticle.

Overall, SERS-based biosensors demonstrate the highest sensitivity, while colorimetric sensors offer simplicity and practicality for portable testing, and fluorescence biosensors provide dynamic real-time monitoring. Notably, nanomaterials, specifically gold/silver nanoparticles, are widely used in optical biosensor fabrication for illicit drug detection.^28,29,43,44,51,52,93,100^ Those biosensors modified with nanomaterials hold lower LOD than those without.

Mao et al. developed a simple, label-free, and cost-effective colorimetric biosensor for MAMP detection based on a G-quadruplex–hemin DNAzyme molecular beacon (DNAzyme MB) and a MAMP-specific aptamer. The sensor achieved a low detection limit of 0.5 nM with a linear range from 8 to 500 nM, and demonstrated high selectivity against 15 other common illicit drugs. In real urine samples, the biosensor yielded recovery rates above 85% and showed good agreement with HPLC-MS/MS measurements.^94^ This study developed a surface-enhanced Raman scattering (SERS) sensor based on a glass nanofibrous paper substrate decorated with gold@silver core–shell nanoparticles (Au@Ag), achieving ultrasensitive detection of methamphetamine in water with a detection limit as low as 7.2 ppt (Figure 5a).^53^ Mao et al. reported an Au-aptamer-based biosensor for colorimetry detection of MAMP and cocaine and evaluated its ability in wastewater samples (Figure 5b).^28^ The average recoveries of MAMP and COC in spiked effluent wastewater samples were 85.5% and 83.9%, respectively, when comparing the measured concentrations with the actual concentrations. These results demonstrate the strong potential of this biosensor for multiplexed detection of illicit drugs in wastewater, supporting its application in community-level drug consumption assessment.

**Figure 5:**
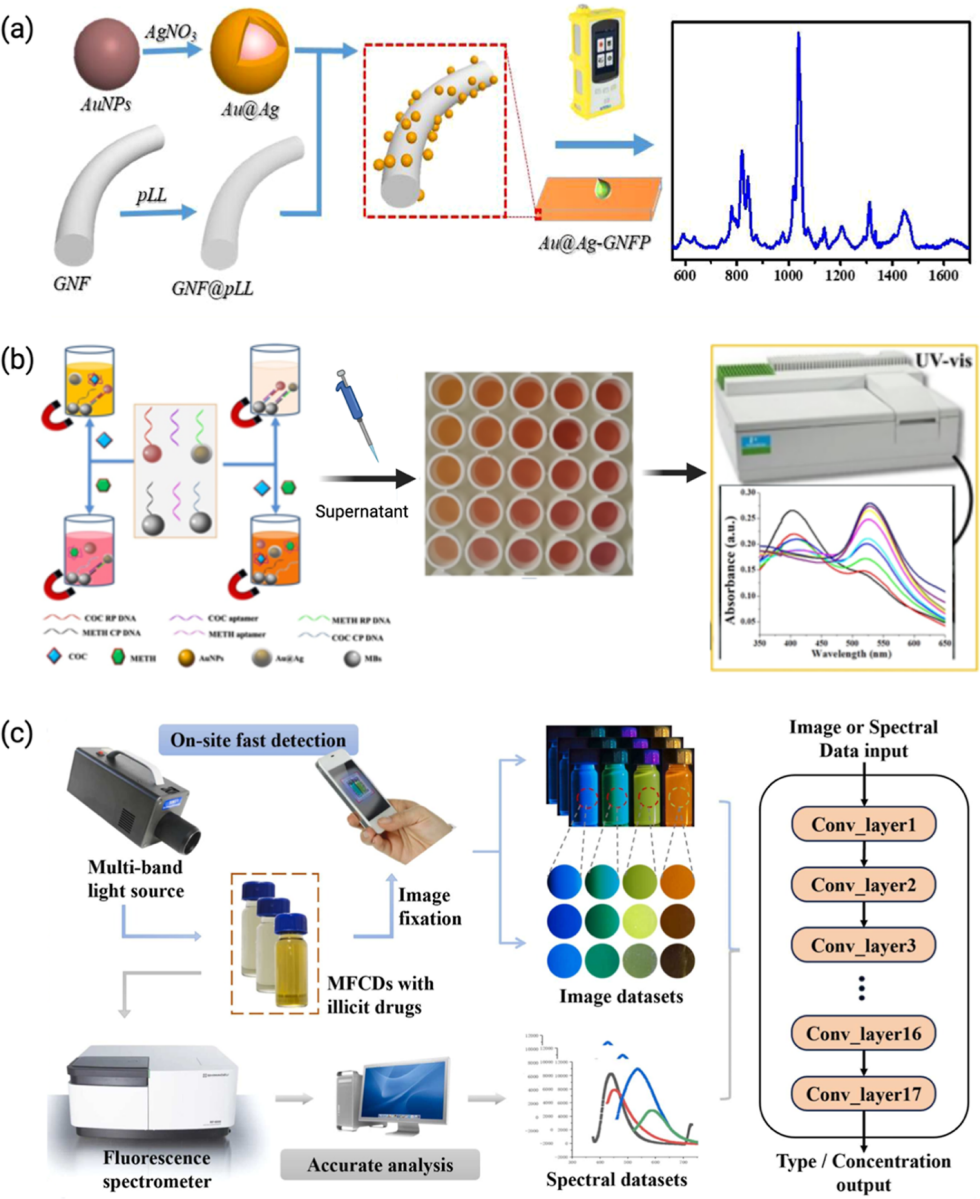
(a) Schematic illustration of the self-assembly of Au@Ag on a glass nanofibrous paper (GNFP) SERS substrate for colorimetry detection of MAMP and cocaine.^53^ Reproduced from ref 53 under the Creative Commons CC-BY license. Copyright 2021 Springer. (b) Schematic illustration of colorimetric detection of MAMP and cocaine based on nonaggregated nanoparticles (C-RP: cocaine reporter probe, M-RP: methamphetamine reporter probe, M-CP, methamphetamine capture probe, and C–CP: cocaine capture probe).^28^ Reproduced with permission from ref 28. Copyright 2019 Elsevier. (c) Flowchart of dual-modal identification of illicit drugs by multicolor fluorescent carbon dots (MFCDs) and deep learning.^101^ Reproduced with permission from ref 101. Copyright 2023 Elsevier.

Biosensor-based techniques present a compelling alternative to traditional analytical methods for illicit drug detection in WBE. While established techniques like GC–MS and LC–MS offer exceptional sensitivity and robust identification, they require complex sample preparation, expensive instrumentation, and specialized personnel, making them less practical for large-scale or real-time surveillance.^30,34,37,79,102,103^ In contrast, biosensors offer a combination of high sensitivity, selectivity, and fast analysis with simplified operational procedures, making them more suitable for on-site and field applications. For instance, biosensors have achieved detection limits as low as 7.2 ng L^–1^ for MAMP and 1000 ng L^–1^ for cocaine, comparable to traditional methods yet with the added benefit of portability and lower operational costs.^28,53^ Despite certain limitations such as biocomponent degradation and potential interference from complex sample matrices, biosensors stand out as a practical and scalable solution for WBE. Their ability to provide near-instantaneous results without the need for extensive sample pretreatment highlights their potential in enhancing real-time surveillance of community-level drug consumption patterns.

Additionally, while some biosensors (e.g., enzymatic^18,23,60^ ) are designed for single-use due to fouling or loss of bioactivity, other formats such as electrochemical aptasensors^24,26,54,68,69,73,74^ or MIP-based sensors^65,66,81,104^ can be regenerated and reused multiple times depending on surface chemistry and regeneration protocols. Certain biosensor platforms, especially those based on electrochemical arrays,^23,105,106^ or surface-enhanced Raman scattering (SERS),^107,108^ have demonstrated potential for simultaneous detection of multiple analytes. This offers a promising advantage over traditional LC–MS workflows, which often require multiple runs or transitions.

## 4 Biosensors for Wastewater-Based Illicit Drug Surveillance

Electrochemical and optical biosensors have been used to detect illicit drugs in wastewater samples. The general workflow includes sample collection and preparation, where wastewater is collected from sewage treatment plants or specific locations of interest (e.g., nightlife districts, music festivals, universities), followed by filtration and preconcentration if needed. Biosensors then use specific biological elements to capture illicit drugs such as cocaine, MAMP, THC, fentanyl etc., triggering a measurable signal transduced into an optical or electrochemical response. Electrochemical biosensors convert molecular binding events into current, voltage, or impedance changes, while optical biosensors detect changes in light absorption, fluorescence, or surface plasmon resonance. Finally, the detected concentrations are used to estimate per capita drug consumption, applying wastewater flow rates, drug metabolism rates, and population normalization factors.

A comparison of biosensors with conventional WBE methods shows that biosensors can achieve comparable or superior sensitivity, with SERS and electrochemical biosensors reaching fM–pM levels, while LC–MS typically operates in the ng L^–1^–pg L^–1^ range.^29,30,45^ The time-to-result for biosensors is within minutes to an hour, compared to several hours or even days for LC–MS/GC–MS, making biosensors a much faster alternative. Furthermore, biosensors are significantly cheaper, as they eliminate the need for expensive centralized laboratories and highly trained personnel. Their portability makes them highly suitable for on-site testing, unlike mass spectrometry-based techniques, which require specialized laboratory settings.

However, biosensor-based WBE faces several technical and practical challenges. First, the complex and variable composition of wastewater poses a significant challenge to the performance of biosensor-based WBE platforms. Raw sewage contains a mixture of suspended solids, humic substances, surfactants, salts, heavy metals, and microbial byproducts, all of which can interfere with biosensor signal generation through multiple mechanisms. These include nonspecific adsorption to the sensor surface, masking or denaturation of biorecognition elements, and electrochemical or optical background noise that obscures low-intensity target signals.

For example, aptamer-based electrochemical sensors may exhibit signal drift or suppression in high-salt or surfactant-rich environments, where the aptamer structure becomes destabilized or the redox tag is shielded.^82,89^ Similarly, SERS biosensors can suffer from hotspot fouling by organic debris, leading to signal quenching and reduced reproducibility. Moreover, fluctuations in pH and ionic strength can alter the binding affinity of recognition elements, further compromising sensor accuracy.^82^

To mitigate matrix interference, several strategies are being developed (though some of them have not been applied in developing biosensors for illicit drug detection). These include prefiltration and solid-phase extraction to remove particulates and matrix modifiers;^109^ surface modifications with antifouling coatings such as polyethylene glycol (PEG),^110^ zwitterionic layers,^59^ or molecular sieves; and ratiometric or internal reference-based signal correction. More advanced approaches involve integrating microfluidic separation modules upstream of biosensor units,^106,111^ or applying machine learning algorithms to distinguish analyte-specific signals from background patterns. Nevertheless, robust performance in unprocessed wastewater remains a key benchmark for future biosensor development.

Another critical barrier to translating biosensor-based WBE into actionable public health tools lies in the lack of standardization, which manifests on two levels: data comparability and protocol harmonization. On the data level, inconsistent reporting formats, including the use of different units for detection limits (e.g., mol/L vs μg/L), nonstandardized calibration models, and inconsistent approaches to peak intensity normalization, make it difficult to compare performance across studies. For example, Raman-based biosensors often report relative intensity changes without internal references, while electrochemical sensors may rely on different redox markers or electrode surface areas, hindering cross-platform comparison. These issues reduce the interpretability of biosensor data and complicate benchmarking against mass spectrometry-based standards.

On the methodological level, variations in sensor fabrication, surface modification chemistry, and sample pretreatment (e.g., filtration, dilution, or solid phase extraction enrichment) contribute to significant reproducibility challenges. Additionally, differences in how spiking and recovery tests (given the condition that few real world tests were conducted) are performed in real wastewater samples often lead to over- or underestimation of biosensor performance. Without harmonized protocols, it is difficult to assess whether a reported detection limit reflects true field applicability or lab-optimized conditions.

To address these issues, future development should align biosensor evaluation methods with existing international guideline or suggestions for WBE, such as those published by the European Union Drugs Agency (EUDA)^112^ or United Nations Office on Drugs and Crime (UNODC),^113^ which provide frameworks for sampling, normalization, and analyte quantification. Establishing consensus on biosensor-specific reporting metrics,^56^ such as “effective LOD/LLOQ in raw wastewater”, “normalized SERS enhancement factor”, or “on-site reproducibility”, will be essential for integrating biosensors into standardized WBE workflows.

Third, translating biosensor information into meaningful, epidemiologically relevant insights remains one of the most critical challenges in WBE. Unlike traditional centralized workflows (such as LC/GC–MS), biosensor networks, especially when deployed in the spots of interest, are expected to generate high-frequency, high-volume data streams from multiple sites. Without scalable interpretation and integration frameworks, the utility of these data is severely limited. This is where Internet of Things (IoT) architectures and artificial intelligence (AI) can play transformative roles.

IoT-enabled biosensor platforms can continuously transmit sensor data, flow rates, and environmental metadata (e.g., temperature, pH, conductivity) to cloud-based servers in real time. These data streams can then be processed by AI models, such as machine learning algorithms (e.g., random forests, long short-term memory (LSTM) networks), to (1) correct for environmental variation and sensor drift; (2) identify anomalous patterns or emerging usage events; (3) fuse biosensor data with auxiliary sources (e.g., weather, flow, public holidays) for better resolution; (4) translate signal data into estimated drug usage metrics using trained regression models.

Recent studies have demonstrated the potential of hybrid sensor-AI systems to achieve automated detection, classification, and quantification of analytes in complex environmental settings.^114^ As biosensors scale into smart city infrastructures, the synergy between sensing technologies, digital connectivity, and intelligent analytics will be central to realizing real-time, actionable WBE. However, these applications are still in early stages and face challenges related to training data availability, model generalizability, and data privacy governance.

Encouragingly, recent efforts have begun to bridge laboratory research and real-world implementation. For instance, Mao et al. reported the successful evaluation of community-wide illicit drug detection in Chinese cities with biosensor-based WBE^53^ (Figure 6).

**Figure 6:**
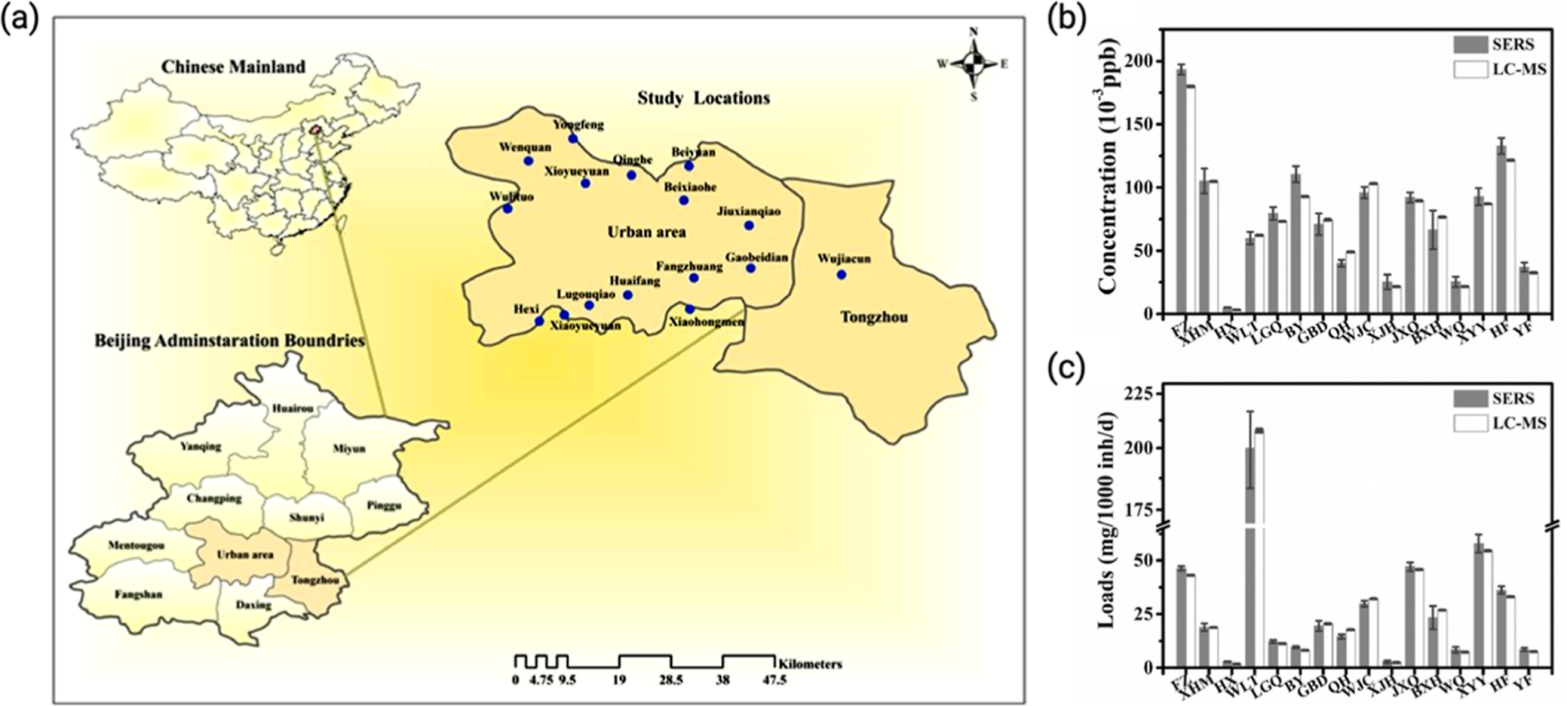
(a) Locations of the 16 wastewater treatment plants in the urban area of Beijing. The study locations were abbreviated as follows: FZ, Fangzhuang; XHM, Xiaohongmen; HX, Hexi; WLT, Wulituo; LGQ, Lugouqiao; BY, Beiyuan; GBD, Gaobeidian; QH, Qinghe; WJC, Wujiacun; XJH, Xioajiahe; JXQ, Jiuxianqiao; BXH, Beixiaohe; WQ, Wenquan; XYY, Xiaoyueyuan; HF, Huaifang; YF, Yongfeng. (b) Normalized methamphetamine concentrations in wastewater treatment plants were measured using the SERS sensor (white) and HPLC-MS/MS (black). (c) Estimated average loads of methamphetamine where wastewater samples were collected.^53^ Reproduced from ref 53 under the Creative Commons CC-BY license. Copyright 2021 Springer.

This study demonstrates that biosensor-based WBE can be effectively utilized for large-scale illicit drug monitoring, providing a cost-effective, and scalable approach to assess drug consumption trends at the community level. By integrating advanced biosensing technology with wastewater surveillance, this method offers a noninvasive and objective alternative to traditional drug monitoring strategies, enabling proactive public health interventions, policy evaluation, and environmental risk assessment.

## 5 Conclusion and Perspectives

Illicit drug use remains a persistent and growing challenge worldwide, with significant implications for public health and the environment. WBE presents a transformative approach for illicit drug monitoring, offering a rapid, cost-effective, and scalable solution to address the increasing public health and environmental concerns associated with drug abuse. By combining the broad coverage of WBE with the high sensitivity and selectivity of biosensors, this innovative method has demonstrated promising potential in providing near real-time insights into community drug consumption trends. The successful integration of biosensors into WBE systems has the potential to facilitate evidence-based policymaking, inform harm reduction strategies, and enhance public health interventions. Moreover, by monitoring drug residues and their metabolites in wastewater, biosensor-based WBE also plays a crucial role in evaluating the effectiveness of drug decriminalization policies and rehabilitation programs.

Looking ahead, biosensors are critical to supportstrategic roles in WBE by integrating with emerging digital and urban infrastructure. In particular, embedding biosensor networks into the IoT and smart city frameworks could revolutionize how drug surveillance data are collected and interpreted. As envisioned, numerous sensors deployed throughout a city’s wastewater network would continuously transmit data to cloud-based platforms, enabling real-time mapping of drug consumption patterns across neighborhoods. This convergence of biosensing technology, wireless connectivity, and intelligent analytics would create a dynamic surveillance system capable of spotting spikes or shifts in drug use as they happen. For example, multimodal models could combine live biosensor readings with wastewater flow rates, weather, and demographic insights to better contextualize the findings. Machine-learning algorithms (from random forests to LSTM networks) have already been applied to retrospectively analyze WBE data; feeding them with real-time biosensor outputs promises to enhance the temporal resolution of these models, improving event responsiveness and extending coverage into areas or timeframes where traditional sampling is impractical. The integration of biosensors with IoT and AI-driven data fusion and machine learning paves the way for an intelligent WBE system that not only detects drug trends faster, but also situates those trends in space and time.

In parallel, AI also holds promise for improving biosensor performance and data interpretion. Neural networks and other learning models can be trained to fit the environmental variability, extract patterns from noisy data, and compensate for matrix-induced distortions which is particularly important for sensors operating in raw or minimally treated wastewater. These capabilities may significantly enhance the accuracy and robustness of field-deployed biosensors.

However, the practical intelligent biosensing ecosystems will require the creation of shared, annotated biosensor data sets, standardized evaluation protocols, and cross-disciplinary collaboration. Integrating sensing hardware, cloud-based data management, and AI interpretation pipelines will be essential for moving from prototype to scalable, public health-ready systems.

Ultimately, the future of biosensor-enabled WBE depends not only on technological innovation, but also on system-level integration, data interoperability, and ethical governance. These elements, when aligned, can transform biosensor networks into real-time, adaptive platforms for evidence-based drug policy, early intervention, and public health protection.

## Data Availability

This review article does not report any original data. All data discussed and analyzed in this review are derived from previously published studies, which are appropriately cited within the text. No new data sets were generated or analyzed during the current study.
